# The Sunflower WRINKLED1 Transcription Factor Regulates Fatty Acid Biosynthesis Genes through an AW Box Binding Sequence with a Particular Base Bias

**DOI:** 10.3390/plants11070972

**Published:** 2022-04-02

**Authors:** Rosario Sánchez, Irene González-Thuillier, Mónica Venegas-Calerón, Rafael Garcés, Joaquín J. Salas, Enrique Martínez-Force

**Affiliations:** 1 Instituto de la Grasa (CSIC), Pablo de Olavide University Campus, Building 46, Carretera de Utrera km 1, 41013 Seville, Spain; rsanchez@ig.csic.es (R.S.); irenegonthu@gmail.com (I.G.-T.); mvc@ig.csic.es (M.V.-C.); rgarces@ig.csic.es (R.G.); jjsalas@ig.csic.es (J.J.S.); 2 Jealotts Hill International Research Centre, Bracknell, Berkshire RG42 6EY, UK

**Keywords:** *Helianthus annuus*, fatty acid biosynthesis, WRINKLED1 (WRI1) transcription factor, AW box binding sequence, electrophoretic mobility shift assay

## Abstract

Sunflower is an important oilseed crop in which the biochemical pathways leading to seed oil synthesis and accumulation have been widely studied. However, how these pathways are regulated is less well understood. The WRINKLED1 (WRI1) transcription factor is considered a key regulator in the control of triacylglycerol biosynthesis, acting through the AW box binding element (CNTNG(N)_7_CG). Here, we identified the sunflower *WRI1* gene and characterized its activity in electrophoretic mobility shift assays. We studied its role as a co-regulator of sunflower genes involved in plastidial fatty acid synthesis. Sunflower WRI1-targets included genes encoding the pyruvate dehydrogenase complex, the α-CT and BCCP genes, genes encoding ACPs and the fatty acid synthase complex, together with the *FATA1* gene. As such, sunflower WRI1 regulates genes involved in seed plastidial fatty acid biosynthesis in a coordinated manner, establishing a WRI1 push and pull strategy that drives oleic acid synthesis for its export into the cytosol. We also determined the base bias at the N positions in the active sunflower AW box motif. The sunflower AW box is sequence-sensitive at the non-conserved positions, enabling WRI1-binding. Moreover, sunflower WRI1 could bind to a non-canonical AW-box motif, opening the possibility of searching for new target genes.

## 1. Introduction

Plant oils are important elements of human diet, as well as for industrial applications (e.g., detergents and lubricants) and biodiesel production. As the global demand for plant oils is rapidly increasing, reliance on the production of higher plant oils has grown. Many plants synthesize triacylglycerol (TAG) in seeds as an essential means to store and provide energy for seedling development [1]. TAG biosynthesis involves two major steps, fatty acid (FA) biosynthesis and TAG assembly, in which different cell compartments participate: plastids, the cytosol and the endoplasmic reticulum [2]. FA and TAG synthesis are regulated by different factors in the different cellular compartments of plants, and each reaction is catalyzed by specialist enzymes [3].

The sugars derived from photosynthesis in source tissues are imported into the developing seeds and converted into FA precursors in the cytosol by glycolysis [4,5]. De novo FA biosynthesis takes place in the chloroplasts of plant vegetative tissues or in the plastids of non-photosynthetic ones. The metabolic pathways driving FA synthesis have been extensively studied in plants (for a review see [3]). Briefly, the pyruvate dehydrogenase complex (PDC) generates acetyl-CoA, the building block for FA production. FA biosynthesis begins with the formation of malonyl-CoA from acetyl-CoA driven by the heteromeric acetyl-CoA carboxylase (ACC). The malonyl group of malonyl-CoA is then transferred to an acyl-carrier protein (ACP), forming malonyl-ACP. Acyl chains are produced by the fatty acid synthase (FAS) complex, which uses acetyl-CoA as a starting unit, while malonyl-ACP provides the two carbon units required for chain elongation. Acyl chains are ultimately desaturated and/or hydrolyzed by stearoyl-ACP desaturase (SAD) and acyl-ACP thioesterases (FAT), respectively, releasing the free FA.

The use of DNA microarrays and transcriptome analyses has provided detailed information regarding the expression of genes involved in plant metabolic processes such as FA biosynthesis [6,7]. It appears that a number of the genes encoding core enzymes involved in FA synthesis are likely to be transcriptionally co-regulated [8]. For instance, the induction of these genes is coordinated in embryonic tissues at the onset of seed maturation to ensure high levels of oil storage [9,10,11]. Transcription factors or proteins regulating mRNA turnover can control these changes in expression and indeed, genetic studies in Arabidopsis have revealed some of the factors that control seed oil biosynthesis. As such, several steps of FA synthesis are regulated by WRINKLED1 (WRI1) [10,12,13,14], a gene under the direct control of the transcription factor LEAFY COTYLEDON2 (LEC2) [12], along with LEC1, FUSCA3 (FUS3) and ABA INSENSITIVE3 (ABI3), considered to be a key regulator of seed development [15].

In Arabidopsis, the *WRI1* gene encodes a transcription factor of the large APETALA2/ethylene-responsive element binding protein (AP2/EREBP) family [16]. Loss-of-function Arabidopsis mutants have no obvious phenotype during vegetative development, but they produce wrinkled, incompletely filled seeds, with an 80% reduction in seed oil content [17]. In addition, these Arabidopsis *wri1* mutants suffer a delay in embryo elongation and a modification of seed oil FA composition toward longer and more desaturated FAs [12]. A combination of molecular and biochemical approaches identified possible WRI1-binding motifs in certain target genes [13,14], and based on promoter sequence comparisons, the CNTNG(N)_7_CG motif was identified as the consensus Arabidopsis WRI1 binding site and designated as the AW box [14]. WRI1 target sequences are found upstream of genes encoding for enzymes involved in glycolysis (sucrose synthase, pyruvate kinase and pyruvate dehydrogenase -PDH), subunits of ACC (biotin carboxyl carrier protein 2 -BCCP2- and its biotin attachment domain containing -BADC- homolog, carboxyltransferase -CT- and biotin carboxylase -BC), components of the FAS complex (malonyl-CoA:ACP malonyltransferase -MCMT, ketoacyl-ACP synthase -KAS, hydroxyacyl-ACP dehydrase -HAD, enoyl-ACP reductase -ENR- and ACPs), SAD, oleoyl-ACP thioesterase and genes involved in lipoic acid synthesis, a cofactor of PDH [10,12,14,18,19,20,21,22,23,24]. More than 20 WRI1 target genes have been identified by comparing gene expression in wild type (WT) plants with that of *wri1* mutants and WRI1 overexpressing lines, and WRI1 promoter binding was confirmed in electrophoretic mobility shift assays (EMSA) and microscale thermophoresis experiments. The distance between the AW box and the translation initiation site (TIS) strongly influences the role of the AW box [20], and the majority of active AW sites lie less than 200 bp from the TIS in true WRI1 target genes [14,20].

*WRI1* orthologs have been identified in many plant species, including *Brassica napus* [25], *Zea mays* [26], *Elaeis guineensis* [27], *Brachypodium distachyon* [28], *Glycine max* [29] and *Oryza sativa* [30]. The features of the *WRI1* gene, its transcriptional regulation and post-translational protein modifications have been studied widely in Arabidopsis, and in other dicotyledonous and monocotyledonous plants [31,32,33]. Sunflower (*Helianthus annuus* L.) is an important oilseed crop cultivated worldwide. Like many other oilseed crops, several attempts to modify its oil composition have been made, for example, the development of new sunflower cultivars from mutagenized seeds with increased saturated FA levels [34]. The biochemical pathways leading to oil synthesis and accumulation in sunflower seeds have also been studied widely [35,36]. Analytical, biochemical, molecular and spatio-temporal expression studies [37,38,39,40,41,42,43,44,45,46,47] have enabled FA and TAG synthesis to be modelled in sunflower seeds, even though the regulation of these processes is less well characterized in sunflower. Recently, H3K4me3 epigenetic modifications were defined and related to the expression of FA-related genes, and of the VIV1 (homologous to Arabidopsis ABI3) and FUS3 transcription factors in developing sunflower seeds [48].

In this study, we identified the sunflower *WRI1* gene and characterized its binding activity. We sought not only to study its role as a co-regulator of sunflower genes involved in plastidial FA synthesis, identifying the genes recognized and bound by this transcription factor, but also to decipher the clues in terms of sequence that enable sunflower WR1 to discriminate between 14 bp consensus AW boxes and choose which to bind to. Interestingly, WRI1 mainly regulates genes from the very early steps of plastidial FA synthesis, particularly influencing the PDC subunits and the ACC complex, as well as acting through key FAS genes, driving the synthesis of oleic acid via *FATA1* gene regulation and establishing a push–pull strategy. We also analyzed the sequence of the functional sunflower AW box motif to determine if there is any base bias at the N positions of the sunflower AW box that drives active transcriptional regulation. As a result, we concluded that the sunflower AW box is sequence-sensitive at these non-conservative N positions.

## 2. Results

### 2.1. The Sunflower Genome Contains One Single WRI1 Gene

The *A. thaliana* WRI1 protein (430 amino acids), encoded by the *At3g54320.1* gene (UniProtKB/Swiss-Prot Acc. Number Q6X5Y6.1; https://www.ncbi.nlm.nih.gov/protein/Q6X5Y6. Accessed on 28 January 2021), was used as the query to search for *H. annuus* homologs in a public database (Heliagene) using the BLASTP algorithm. One particular protein sequence was detected with both the lowest E-value (2.63 × 10^−109^) and the longest alignment (382 amino acids), corresponding to the *HanXRQr2Chr14g0649121* gene located on chromosome 14. All of the other homologs found had higher E-values, even for shorter alignments (174 amino acids or less). The percentage identity/similarity between AtWRI1 and the first four homologous sunflower proteins identified confirmed the *HanXRQr2Chr14g0649121* gene to be the sole homolog of *AtWRI1*, with 50% identity and 64% similarity as opposed to less than 37% identity and 47% similarity for the other three proteins detected. In addition, only *HanXRQr2Chr14g0649121* was expressed strongly in seeds, while the other three genes were preferentially expressed in leaves (Heliagene). Accordingly, we considered the *HanXRQr2Chr14g0649121* gene to be the *H. annuus* L. WRINKLED1 gene, and we refer to it hereafter as *HaWRI1*.

**Figure 1:**
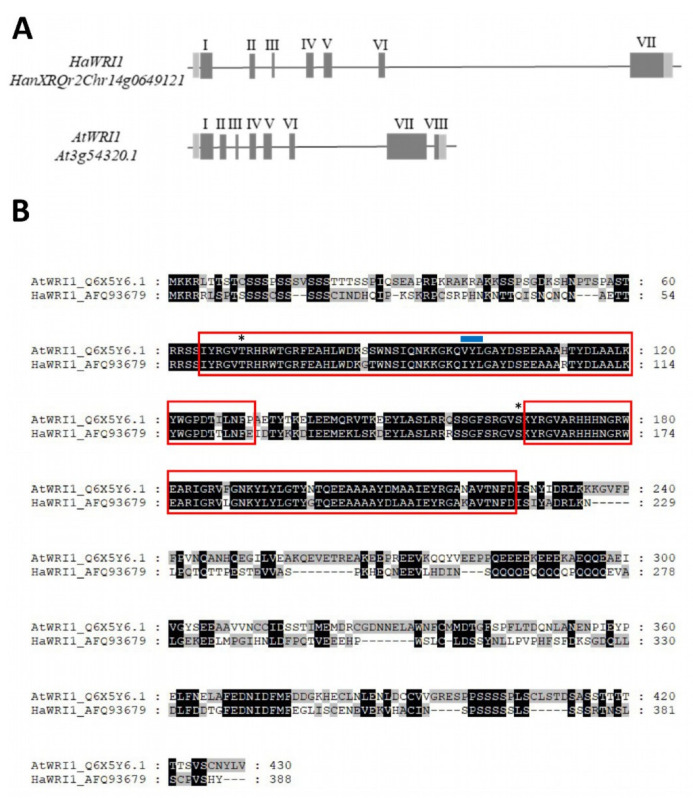
Comparison of the gene structure and protein sequence of WRINKLED1 in *Helianthus annuus* and *Arabidopsis thaliana*. (**A**) Arabidopsis (*AtWRI1*) and sunflower WRINKLED1 (*HaWRI1*) gene structures. UTR: light grey boxes; EXON: dark grey boxes; and Exon number: roman numerals. (**B**) AtWRI1 and HaWRI1 protein alignment. The AP2-EREBP DNA binding domain is highlighted by a red box, and the asterisks mark the sites in AtWRI1 phosphorylated by KIN10 (T70 and S166). The VYL domain encoded by 9 bp exon 3 is marked with a blue line. The Acc. numbers are shown on the left.

This *HaWRI1* gene shared the similar exon–intron structure of the *AtWRI1* gene (Figure 1A), which included the 9 bp exon 3 that encodes the VYL transcriptional activation motif [27], although a single base change (G/A) turned this motif into IYL in HaWRI1. The highly conserved AP2 domain spanning all exons was also conserved in HaWRI1. The corresponding *HaWRI1* cDNA (1167 bp) from the CAS−6 sunflower line was cloned (see Methods) and its sequence was verified prior to depositing it in GenBank with the accession number JX424422.1. HaWRI1 resulted in a protein 3 amino acids shorter (388) than that predicted in the Heliagene database, probably due to the distinct sunflower background (HA412-HO). Nevertheless, it included the main features of the WRI1 transcription factor (see Figure 1B for an alignment of the AtWRI1 and HaWRI1 proteins), the two AP2 domains (59–125 and 161–219 amino acids) and the two phosphorylation sites known to be involved in the protein stability (T64 and S160) [31].

### 2.2. Sunflower WRI1 Binds to the AW Box Motif Present in the Acyl-ACP Thioesterase FATA1 Gene

The primary function of WRI1 in seed oil deposition appears to be the positive regulation of genes that encode enzymes involved in late glycolysis and FA biosynthesis [11]. The functionality of HaWRI1 as a transcriptional activator was tested in EMSAs using DNA fragments containing the consensus AW box binding motif CNTNG(N)_7_CG [14]. To that end, we first cloned the DBD of HaWRI1, fused it to thioredoxin (TRX) to improve its solubility and expressed it heterologously in *E. coli*, obtaining the 6-His-TRX-DBD recombinant protein (HaWRI1_DBD). Moreover, we also expressed the 6-His-TRX and 6-His-TRX-GFP fusion proteins heterologously to use as negative binding controls in the EMSAs. All of these recombinant proteins were purified by Ni-NTA affinity chromatography as described in the Methods (Figure S1).

We next searched for candidate genes containing an AW box motif in their promoter regions (as described by Maeo et al. [14]). We initially chose to work with FAT and SAD genes, not only due to their involvement in FA biosynthesis in seed plastids but mainly based on the *FATA* gene up-regulation in plants overexpressing WRI1 [14,21]. Two sunflower FAT genes, *FATA1* and *FATB1*, have been described in the literature with different substrate selectivity [40,49,50], as well as two SAD genes, *SAD6* and *SAD17* [49,51]. We searched for the DNA promoter regions of each of these genes in the public sunflower database (Heliagene) using the BLASTN algorithm and the cDNA sequence of each gene as a query. Subsequently, we designed primers to amplify at least 1 kb of the promoter region for each gene, verifying the DNA fragments obtained by sequencing, and used the PlantPAN 3.0 free software to search for the presence of the AW box (Table S2). We found AW box motifs in the *SAD17*, *FATA1* and *FATB1* genes but not in *SAD6*. However, only FATA1 presented an AW box motif in the upstream region close to the ATG codon, −110/−97 bp from the TIS and in the 5′-untranslated region (5′-UTR), as described in Arabidopsis genes positively regulated by WRI1 [14,20]. All of the AW boxes found, their sequences and locations from the TIS are detailed in Table 1, including a sequence similar to an AW box found in *SAD6* but with an extra base, together with the overlapping DNA fragments designed for EMSAs spanning the cloned promoter regions for each of the genes studied (Table S3).

**Figure 2:**
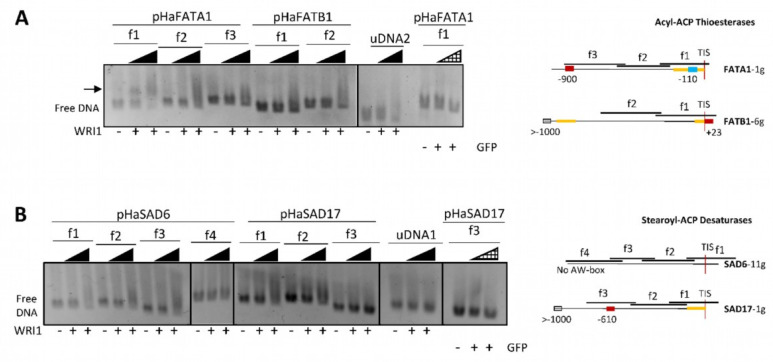
WRINKLED1 binding to the promoter regions (pHa) of sunflower (**A**) acyl–ACP thioesterase (FAT) and (**B**) stearoyl–ACP desaturase (SAD) genes in agarose EMSAs. The arrow indicates the DNA shifted due to WRI1 binding. WRI1, 6–His–TRX–WRI1_DBD fusion protein (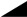 160–640 ng); GFP, 6–His–TRX–GFP fusion protein (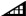 160–640 ng); uDNA1, non–specific DNA1 (HacPGK2); uDNA2, non–specific DNA2 (HaCWI3). Overlapping DNA fragments (f1 to f4, 200–300 ng) tested in each gene in agarose EMSA are detailed on the right. The boxes indicate the AW box motifs, and the numbers indicate the distance from the ATG codon (bp). Binding is indicated as negative (red boxes) or positive (blue boxes), while a colorless AW box means it was not tested. The 5′–UTR is in orange. The sunflower gene names are based on previous publications, and they are followed by the number indicating the chromosome location. TIS, Translational Initiation Site (ATG).

EMSAs were performed to test the binding of the purified HaWRI1_DBD to each overlapping DNA fragment of around 200–300 bp from the sunflower FAT and SAD promoter regions. This fragment size allowed us to resolve the potential shifted bands by electrophoresis in agarose gels. Binding of HaWRI1_DBD to each DNA fragment from the promoter regions of the sunflower *FATA1* and *FATB1* genes (pHaFATs: Figure 2A), and the *SAD6* and *SAD17* genes (pHaSADs: Figure 2B), was analyzed and correlated to the locations of the AW box motifs in the overlapping DNA fragments. As expected, the *FATA1* AW box at −110 bp from TIS included in fragment 1 (*pHaFATA1-f1*, 323 bp) bound to the HaWRI1-DBD, migrated slower in the gels and increased in intensity as the amount of assayed protein was augmented, while the free DNA decreased concomitantly. Moreover, the shift of this fragment was not observed when *pHaFATA1-f1* was incubated with the GFP protein instead of WRI1. The mobility of none of the other DNA fragments tested was seen to alter in the gels, whether or not they contained an AW box motif, neither was that of the *SAD6* sequences with an extra base (*SAD6-f4*) nor the specific binding control reactions with non-specific DNA, or when the GFP protein was used instead of WRI1.

**Figure 3:**
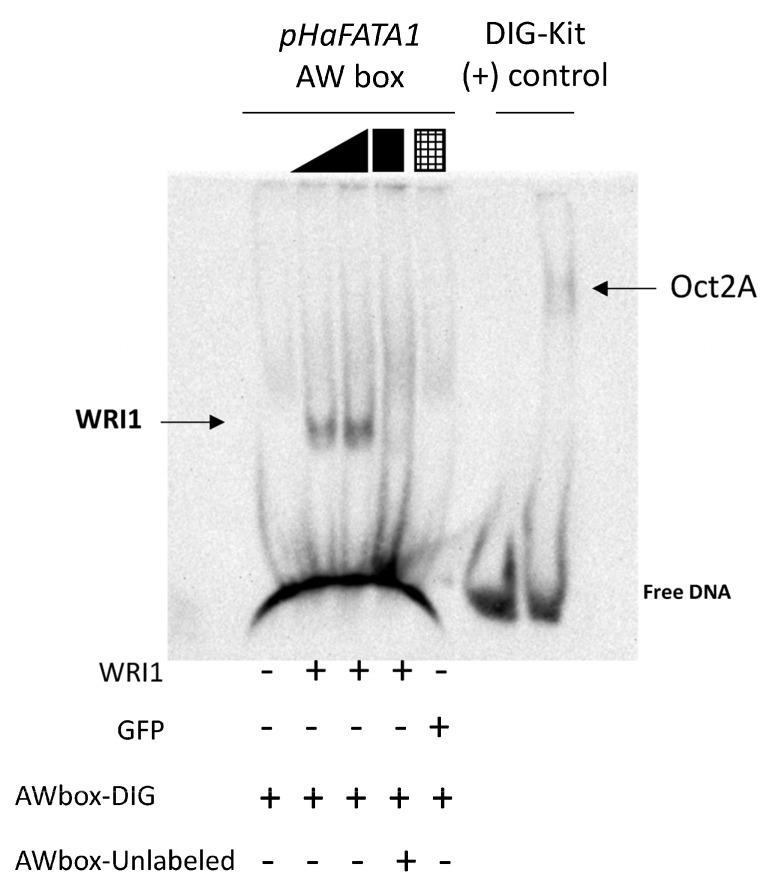
Binding of the HaWRI1_DBD to the AW box motif in the sunflower *FATA1* promoter region (*pHaFATA1*) as evident in digoxigenin (DIG) labelled EMSA. The arrow shows WRI1 or Oct2A (positive–binding control) shifted DNA. The DIG–labelled or unlabelled (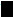 cold competitor reaction) dsDNA (24 bp, 30 fmol) contains the 14 bp AW box motif. WRI1, 6–His–TRX–WRI1_DBD fusion protein (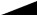 500–1000 ng); GFP, 6–His–TRX–GFP fusion protein (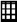 1000 ng); Oct2A, Octamer–binding factor 2A (75 ng).

To assess whether sunflower WRI1 bound specifically to the 14 bp AW box motif present in *pHaFATA1-f1* and not to any other sequence in the 323 bp f1 fragment, we performed EMSAs in acrylamide gels with digoxigenin-labelled DNA incubated with the sunflower WRI1 DBD. The HaWRI1-DBD bound specifically to double-stranded DNA (dsDNA) fragments of 24 bp that contained the 14 bp f1 AW box motif from *pHaFATA1-f1* (the extra random bases were added to give a minimum 5 bp context at each end of the AW box motif, see methods). This shifted band was observed only in the presence of WRI1 and not when the DNA fragment was incubated with GFP or in competition assay with unlabelled dsDNA (Figure 3). Moreover, when HaWRI1-DBD binding to the *pHaFATA1-f1* fragment lacking the 14 bp corresponding to the AW box (*f1-ΔAW-box*, 299 bp: Table S5) was assessed in these EMSAs, no specific binding of this protein, or of the TRX and GFP fusion proteins, was observed (Figure S2). Hence, sunflower WRI1 specifically bound to the AW box located at −110/−97 bp from the TIS in the 5′-UTR of *HaFATA1* gene.

### 2.3. Sunflower WRI1 Regulates Plastidial Fatty Acid Synthesis Mainly at Early Steps of the Pathway

Having confirmed the functionality of HaWRI1 by *in vitro* binding to the AW box motif located in the *FATA1* upstream region, we investigated which other genes involved in sunflower plastidial FA biosynthesis might be regulated by WRI1 in a coordinated manner. In this regard, we were intrigued by how WRI1 could discriminate the canonical AW box motifs also present in the DNA fragments *pHaFATA1-f3*, *pHaSAD17-f3* and *pHaFATB1-f1*, failing to bind to them in the *in vitro* binding assays (Figure 2).

We used the BLASTN algorithm to search for the sunflower homologs of each gene involved in the plastidial FA biosynthetic pathway in the public sunflower database (Heliagene, recently updated in 2020), using the corresponding Arabidopsis genes as queries (Table 2). The Arabidopsis gene sequences were obtained from The Arabidopsis Information Resource (TAIR) in accordance with the Arabidopsis acyl-lipid metabolism pathways (ARALIP). We assessed the existence of AW box motifs in the upstream regions of these genes using the PlantPAN 3.0 free software. We also employed two free online applications to predict the subcellular localization of all of the proteins encoded by these genes (see Materials and Methods) and as expected, most of them were plastidial proteins. The genes with no AW box in their upstream regions or encoding proteins with a subcellular localization other than plastid were ruled out for further study. The genes retained were referred to by their sunflower homologous gene name, the Heliagene ID, and for those with one or more AW boxes, a shorter name was employed (the function acronym followed by a number with the chromosome location, e.g., *BCCP−16g*: Table 2). To be considered as a gene that could be regulated by WRI1 in the seed, the expression of each sunflower gene homolog in the seed was also analyzed based on the transcriptome data in the Heliagene database. According to this database (sunflower cv. HA412-HO), most genes were expressed in seeds, and some of them strongly, such as the *β-PDH−16g*, *BCCP−9g*, *KASI−17g*, *KASIII−5g* and *KAR−17g* genes (Table 2). The seed expression data for all of these genes were indirectly confirmed in the sunflower CAS−9 line as accessible chromatin in ChIP-seq experiments [48]. Bibliographic references are given for those sunflower genes whose expression has already been described in CAS−6 sunflower seeds (Table 2). The expression of the other genes in seeds of the CAS−6 line, such as the *FATB−9g* and genes of the sunflower ACC complex, was also confirmed (data not shown).

For all of the genes expressed in seed that contained one or more AW boxes, we situated the AW box motif in the upstream region relative to the TIS and determined if they fell within the 5′-UTR (Heliagene, see Table 3). We selected all of the genes with AW boxes in 5′-UTR for further EMSAs, designing primers for PCR amplification and sequencing of each DNA fragment containing an AW box motif to use them in EMSAs (Tables S4 and S5). As we wanted to test whether WRI1 could discriminate the consensus binding motif by its sequence alone, we included some AW box motifs found in upstream regions outside the 5′-UTR but close to the TIS (up to −500 bp) in the EMSA studies, and others found far from TIS (up to −2500 bp: see the genes, sequences and positions of all of the AW boxes used in agarose gel EMSAs with sunflower WRI1 in Table 3). We were able to amplify and study all of the selected AW box motifs, except for that in the *LT−5g* 5′-UTR (according to the Heliagene database), possibly because the sequence of the CAS−6 line’s upstream region differs from that deposited in the database.

**Figure 4:**
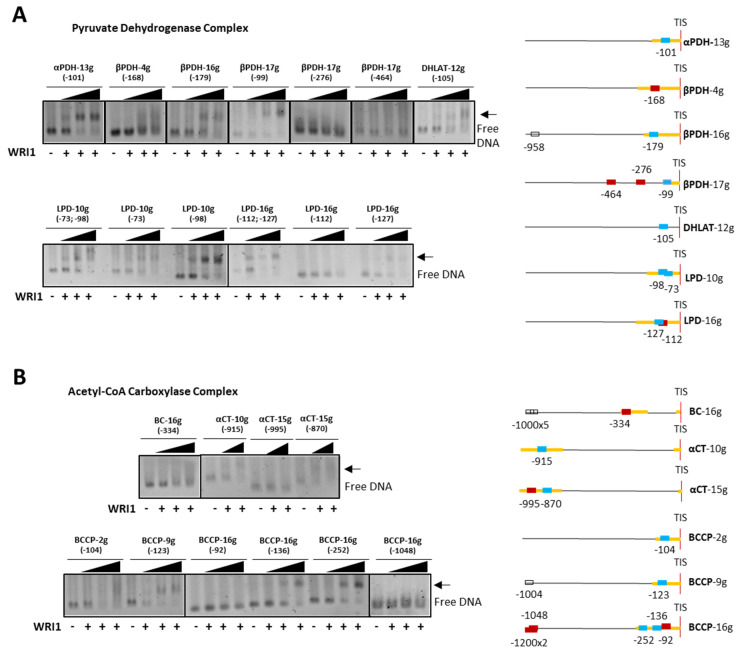
WRI1 binding in agarose EMSA to the sunflower promoter region of genes involved in plastidial fatty acid biosynthesis belonging to pyruvate dehydrogenase (**A**) and acetyl–CoA carboxylase (**B**) complexes. The arrow shows the DNA shift due to WRI1 binding: WRI1, 6–His–TRX–WRI1_DBD fusion protein (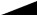 50–150–300 ng). The locations of the AW box motifs relative to the ATG codon in each DNA promoter region analyzed by EMSA (300 ng) are shown on the right. The boxes indicate AW box and the numbers indicate the location from the ATG (bp). Red boxes indicate negative binding and blue for positive binding, while a colorless AW box means it was not tested. The 5′–UTR is in orange. The sunflower gene names are based on previous publications, and they are followed by the number indicating the chromosome location: TIS, Translational Initiation Site (ATG).

The results of the EMSAs with selected medium-size DNA fragments (containing single or multiple AW box motifs) were grouped according to the different enzymatic complexes or proteins that act in plastidial FA synthesis. As such, PDC subunits encoded by the *α-PDH−13g*, *β-PDH−16g*, *β-PDH−17g*, *DHLAT−12g*, *LPD−10g* and *LPD−16g* genes contained an AW box motif in their 5′-UTR that bound the HaWRI1_DBD protein, and that produced a shift in EMSA agarose gels (Figure 4A). The *α-PDH−13g*, *β-PDH−16g* and *DHLAT−12g* fragments each had a single AW box, whereas *β-PDH−17g* contained three boxes at −99 bp, −276 bp and −464 bp, although only that located within the 5′-UTR (−99 bp) gave a positive binding result. *LPD−10g* and *LPD−16g* each contained two AW boxes, both located in the 5′-UTR, yet while both the motifs in *LPD−10g* bound to HaWRI1-DBD, only one motif in *LPD−16g* (at −127 bp) provoked a band shift. When analyzing the AW box motifs from the genes encoding the proteins of the ACC complex, the *BC−16g* and BCCP gene motifs were detected close to the ATG codon, whereas the α-CT genes contained motifs in their 5′-UTR but far from the TIS due to the presence of a 720 bp intron. Only *BCCP−9g* and *BCCP−16g* bound to HaWRI1-DBD, producing a band shift in the assays (Figure 4B). *BCCP−9g* had a single motif (at −123 bp), but *BCCP−16g* contained three motifs near the TIS (at −92 bp, −136 bp and −252 bp), all in the 5′-UTR of the gene. When analyzed individually, the motifs at −136 bp and −252 bp were the only two DNA fragments that clearly underwent a shift in the presence of WRI1.

We also tested AW box motifs located far from the TIS in *BCCP−16g*, 3 motifs at > −1000 bp in the same EMSA, and as expected, no band shift was observed when these were incubated with HaWRI1-DBD. The *BC−16g* AW box was located in the 5′-UTR, and although no definitive band shift was observed, there may have been some weak binding with the greatest amount of protein (smeared lane, Figure 4B, upper panel). Indeed, the *α-CT−10g* and *α-CT−15g* genes contained an AW box in their 5′-UTR with an identical sequence (*αCT−10g* −915 bp and *αCT−15g* −870 bp), which underwent a shift in the EMSAs, whereas no binding to the *αCT−15g* (−995 bp) motif was observed. All of these initial results confirmed that sunflower WRI1 could discriminate between canonical AW box motifs, specifically binding to just some of them in an *in vitro* assay and ruling out those located in the upstream region far from a TIS, even though they might lie in the 5′-UTR. This phenomenon was also observed in the rest of the promoter regions analyzed (see below).

**Figure 5:**
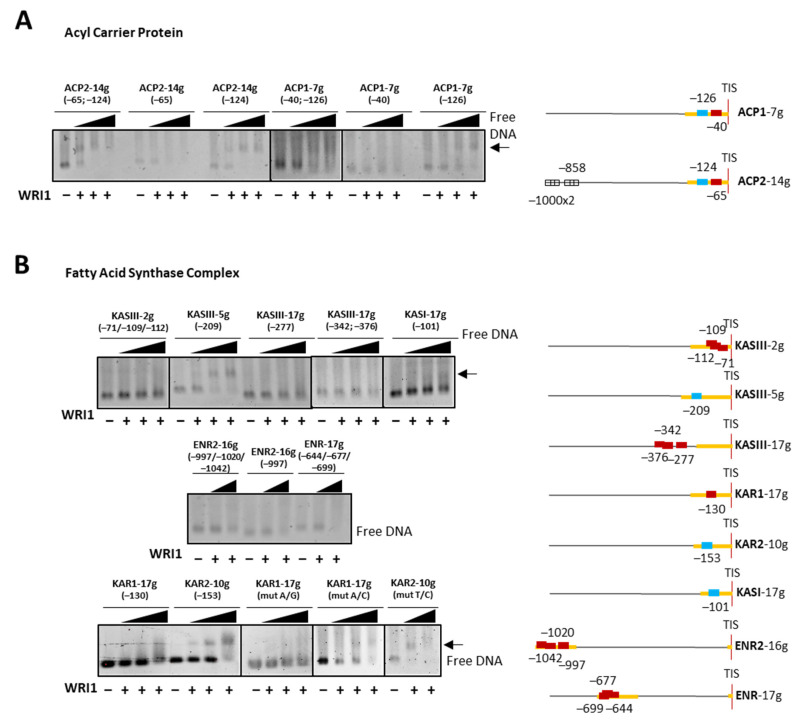
WRI1 binding in agarose EMSA to the promoter regions of sunflower genes involved in plastidial fatty acid biosynthesis, Acyl–carrier proteins (**A**) and those belonging to Fatty Acid Synthase Complex (**B**). The arrow shows DNA shifted by WRI1 binding: WRI1, 6–His–TRX–WRI1_DBD fusion protein (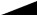 50–150–300 ng). The positions of the AW box motifs relative to ATG codon in each DNA promoter region analyzed by EMSA (300 ng) are shown on the right. The boxes indicate the AW box motifs, and the numbers indicate the positions relative to the ATG (bp). The binding data are indicated as red for negative and blue for positive, while a colorless AW box means it was not tested. The 5′–UTR is in orange. The sunflower gene names are based on previous publications, and they are followed by the number indicating the chromosome location: TIS, Translational Initiation Site (ATG).

HaWRI1_DBD binding to DNA fragments from regions upstream of the sunflower ACP genes was assessed—both *ACP−7g* (*ACP1*) and *ACP−14g* (*ACP2*), which contained two AW box motifs in their 5′-UTR (Figure 5A). Only one AW box motif in each of these two genes produced a band shift, in both cases located a little further away from the TIS (−126 bp in *ACP1* and −124 bp in *ACP2*). Regarding the AW box motifs in the upstream regions of genes belonging to the FAS complex (Figure 5B), we first tested the motifs in selected KASIII genes (*KASIII−2g*, *KASIII−5g* and *KASIII−17g*) and *KASI−17g*. Of these, only the motif located in the 5′-UTR of *KASIII−5g* (−209 bp) produced a band shift when incubated with WRI1. The EMSA DNA fragment from *KASIII−2g* contained three AW boxes in the 5′-UTR, although they were too close together to be analyzed independently, and two of them overlapped and did not bind to WRI1, probably due to this overlap. The *KASI−17g* AW box motif produced a less clean but defined band shift. We also tested motifs located close to the ATG codon but in the 5′-UTR of the *KASIII−17g* upstream region and none bound to the HaWRI1-DBD. Regarding the FAS complex, in addition to the KAS genes, only the KAR and ENR genes contained WRI1-binding motifs. *ENR2−16g* and *ENR−17g* had three AW box motifs, two with an identical sequence (Table 2), and in EMSAs these gene fragments produced smears like *BC−16g* and *BCCP−2g*, suggesting some weak binding (Figure 4). Hence, the sunflower genes targeted by WRI1 were mainly those involved in the early steps of the plastidial pathways studied, including acetyl-CoA and malonyl-CoA synthesis, as all subunits of the PDC were encoded by genes bound by WRI1, along with the α-CT and BCCP genes of the ACC. Further down these pathways, a few key genes were bound by WRI1, such as the genes coding for ACP, KASIII and KASI and the *FATA1* gene, affording the WRI1 transcript a co-regulatory role in sunflower seeds.

### 2.4. Sunflower WRI1 Can Bind to a Non-Canonical AW Box Motif

When considering the KAR genes, the *KAR−17g* (*KAR1*) gene contained a single AW box motif (Table 2) in its 5′-UTR at −130 bp, close to the TIS. Interestingly, the other sunflower KAR gene, *KAR−10g* (*KAR2*), did not have a canonical (CNTNG(N)_7_CG) AW box, but rather, it has an almost identical sequence to that located in *KAR1* in its 5′-UTR (CTTAGATTATATCG: Table 3) that could be considered an “AW box with an extra N base” (CTT**T**AGGTTATATCG). To clarify the role of WRI1 in regulating these two isogenes that share 98.5% identity in their coding sequences and similar seed expression [45], we studied their promoter regions in EMSAs.

We amplified, cloned and sequenced DNA fragments corresponding to the upstream regions of *KAR1* and *KAR2* using a common pair of primers due to the strong identity in their sequence, even in their 5′-UTR (Table S4). The *KAR1* DNA fragment of 279 bp contained an AW box motif −130 bp from the TIS, while the 261 bp *KAR2* fragment contained a sequence similar to the AW box but with an extra base at −153 bp from the TIS. After incubating these two fragments with HaWRI1-DBD, we surprisingly observed a defined band shift in agarose gel EMSA. This *KAR2−10g* band became more intense as the amount of protein increased, whereas a smear developed when *KAR1−17g* DNA was incubated with the maximal amount of protein (Figure 5B, bottom panel). Specific binding control reactions with non-specific DNA and other purified proteins (GFP or TRX) produced negative results in EMSAs (Figure S3). Hence, not only could HaWRI1 bind to a non-canonical AW box motif in vitro, but it also appeared to have a better affinity than to a canonical site in a similar context.

Given the strong identity between the two binding sequences assayed, differing only in one base (adenine at position N_3_ in *KAR1* and guanine in *KAR2*) and the presence of an extra base (thymine) in *KAR2* next to an adenine at position N_2_, we assessed whether the bases at N_3_ or N_2_ were crucial for WRI1 binding. We tested this using three sequences, two for the *KAR1* AW box and one for the *KAR2* motif (Table 3). For *KAR1*, the N_3_ base (adenine) was mutated to a guanine to imitate the *KAR2* AW box at that position (*mut A/G*), or the N_2_ base (adenine) was changed to a cytosine (*mut A/C*) as found in the *FATA1* AW box, also imitating an AW box bound by WRI1 in vitro. For *KAR2*, we mutated the extra base (thymine) adjacent to the N_2_ base to a cytosine (*mut T/C*) due to the presence of a cytosine at N_2_ in most AW boxes bound by sunflower WRI1. The binding of HaWRI1-DBD to these mutated KAR AW boxes (Table S5) was evaluated in agarose gel EMSAs (Figure 5B, bottom panel). The *KAR1 mut A/G* did not undergo a shift with WRI1 despite that mutation producing the same canonical sequence as that in *KAR2* except for the extra base, suggesting that the presence of the extra base in *KAR2* is responsible for the binding of WRI1, and that an adenine at the N_2_ position was not sufficient and the extra base was necessary for binding. However, *KAR1 mut A/C* did bind WRI1, producing a weak but visibly shifted band, reflecting the significant role of a cytosine at position N_2_ as expected. No difference was observed in the *KAR2 mut T/C* AW box compared to the WT, and thus, the presence of an extra base next to the N_2_ adenine seemed to be the only requirement for WRI1 binding.

### 2.5. The Sunflower AW Box Motif That Regulates Transcripts Involved in FA Synthesis Shows Base Bias in Non-Conservative Motifs

**Figure 6:**
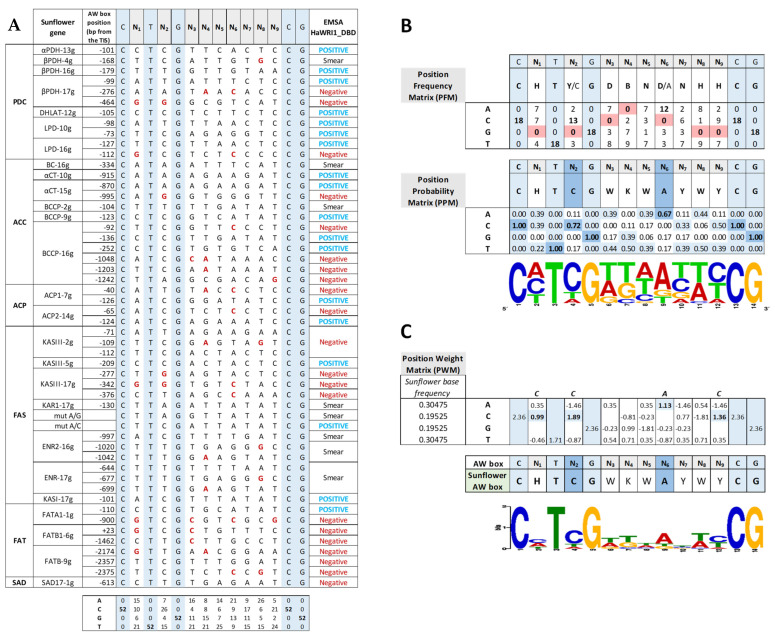
Sunflower AW box sequences present in genes involved in plastidial fatty acid synthesis. (**A**) All of the sunflower AW box motifs (CNTNG(N)_7_CG) analyzed by EMSA for binding to the sunflower WRINKLED1 DNA binding domain (HaWRI1_DBD). The sunflower gene names, according to the functional acronym and chromosome location, and the AW box position relative to the ATG codon (TIS: Translational Initiation Site) are shown on the left. EMSA binding of WRI1 to each sequence is shown on the right and the “forbidden” bases are in red. (**B**) The position frequency and probability matrices (PFM, PPM) for sunflower AW boxes that positively bind WRI1 reveal missing bases (red background) and the base bias (blue background) for most N positions of the canonical sequence. The motif derived from the PPM is shown as a WebLogo pattern. (**C**) The position weight matrix (PWM) gives the sunflower base frequency, and the active sunflower AW box motif is shown as a MEME pattern.

We aligned the 52 canonical AW box motifs analyzed here in EMSAs, classifying them according to their binding by sunflower WRI1 (Figure 6A). To identify the features enabling specific boxes to be recognized by and to bind to sunflower WRI1, we generated a position frequency matrix (PFM) and a position probability matrix (PPM) with the help of the MAST software available in the MEME suite using only the sequences that clearly bound WRI1 in EMSAs—18 of the 52 analyzed. These matrices revealed missing bases and base bias at most non-conservative N positions of the sunflower AW box motif (Figure 6B). Thus, guanine was absent at the N_1_, N_2_, N_8_ and N_9_ positions, adenine was absent at N_4_ and cytosine was not detected at N_3_ and N_6_. These missing bases were considered as “forbidden” bases at those particular positions in the non-conservative part of the consensus AW box (as described by Maeo et al., 2009), suggesting that WRI1 recognizes and binds to the AW box motif in a sequence-sensitive manner in sunflower. Regarding the base bias observed at most N positions, the prevalent bases at the N_2_ and N_6_ positions were cytosine and adenine, respectively. Further evidence of this sequence sensitivity came from the canonical AW box sequences not bound in vitro by sunflower WRI1 in the EMSAs, which all had one or more “forbidden” bases in their motifs (Figure 6A, in red). When only one forbidden base was present, it always involved position N_2_ (*KASIII−17g* −277 bp; *αCT−15g* −995 bp) or N_6_ (*BCCP−16g* −92 bp; *KASIII−17g* −376 bp; or *ACP2−14g* −65 bp), highlighting the role and bias of both these positions. However, there were some exceptions in the absence of a “forbidden” base that could be explained by the presence/combination of one or more bases other than the critical N_2_ and N_6_ bias, such as *BC−16g* (−334 bp: N_2_ = A; N_6_ = T), *BCCP−2g* (−104 bp: N_2_ = T), *KAR1−17g* (−130 bp: N_2_ = A), *FATB−9g* (−2357 bp: N_6_ = G) and *SAD17−1g* (−613 bp: N_2_ = T; N_6_ = G). Moreover, when all of the AW box motif sequences found in the sunflower genes that were not selected for EMSA due to their distance from the ATG codon (see Table 3) were examined, they mostly contained “forbidden” bases, and three of these motifs that did not show them, had one or more bases at the N_2_/N_6_ position not considered among those within the WRI1 bias (*SAD17−1g*, −1159 bp; *KASII−15g*, −1290 bp; and *LS−5g*, −867 bp). 

A new motif pattern was derived from the PPM (Figure 6B), and we wanted to fit this pattern to the sunflower genomic base frequency [52], for which we generated a position weight matrix (PWM) using the MAST software (Figure 6C). The observed base bias in the PWM corresponding to the 18 sunflower motifs produced a sunflower AW box motif that met the consensus motif [14], yet it was fine-tuned to **C**H**TCG**WKW**A**YWY**CG**, highlighting the relevant role of the cytosine and adenine at the N_2_ and N_6_ positions, respectively.

## 3. Discussion

A high degree of cross-species conservation is a hallmark of master transcriptional regulators, as witnessed for WRI1 from *A. thaliana* [16] and its orthologs in different plant species, both monocots and dicots. In most species, there are mainly single *WRI1* genes, except for *Z. mays* [26] and *C. sativa* [53], where two and three isoforms exist, respectively. Here, we identified and studied the *WRI1* gene as a single copy gene in the genome from an important oil crop plant such as *Helianthus annuus* (*HaWRI1*). The *HaWRI1* gene and protein are consistent with those previously described for *AtWRI1* and its orthologs [31]. However, it was notable that the third exon of *HaWRI1* encodes an IYL domain instead of the expected VYL due to a single (G/A) base change, also evident in some other WRI1s such as the tomato SlWRI1 protein (XP_004231231; https.//www.ncbi.nlm.nih.gov/protein/XP_004231231. Accessed on 3 November 2021). The VYL domain is a highly conserved domain, and site-directed mutagenesis of this motif in AtWRI1 failed to restore the oil content in the *wri1* mutant [27]. However, *WRI1* orthologs identified in *R. communis* [54] and *O. sativa* [30] encode proteins lacking a VYL motif, yet oil biosynthesis *in planta* was still triggered by activating the expression of WRI1 target genes [30,55]. IYL is thought to be produced through a G to A substitution in the first codon of the motif for “V”, as occurs in HaWRI1. As such, the first amino acid “V” seems to be less important in this VYL motif and can be substituted [27,56].

Some WRI1s are expressed strongly in non-seed tissues, such as fruits (e.g., oil palm WRI1). However, numerous WRI1-like proteins are expressed strongly in developing seeds, closely correlated to *AtWRI1* expression. As expected for the sunflower, based on the sunflower transcriptome data (Heliagene), *HaWRI1* was most strongly expressed in seed tissues. When searching for WRI1 homologs in the sunflower database using *AtWRI1*, other putative WRI genes were found with higher expression levels in non-seed tissues (leaves, ligule), probably members of the sunflower WRI gene family whose activity remains to be studied. Up to four members of this family have been described in *A. thaliana* (*AtWRI1−4*) [19], with *AtWRI2−4* expressed in floral organs and active in cutin biosynthesis, and there are three family members in *R. communis* (*RcWRI1−3*) [54], acting in membrane lipid synthesis in vegetative organs.

**Figure 7:**
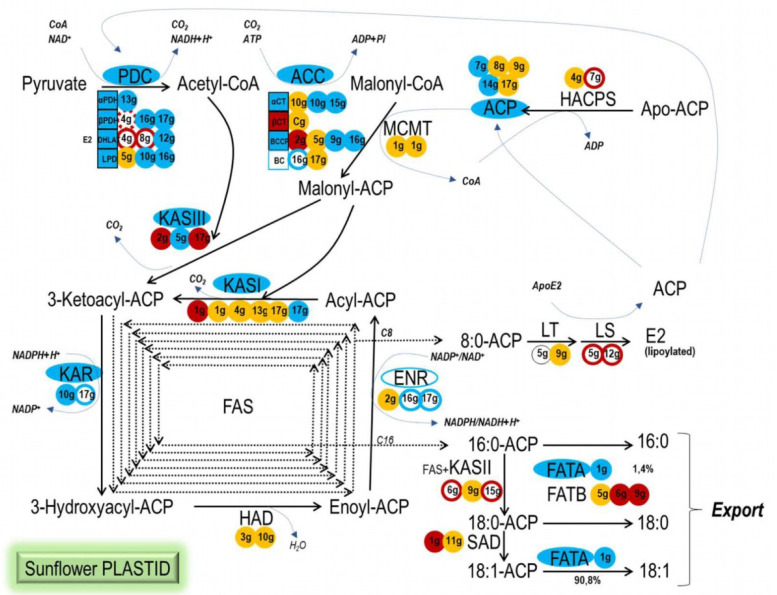
Sunflower WRI1 target genes in the seed plastidial fatty acid biosynthetic pathway. WRI1 regulation of the sunflower gene isoforms studied here in EMSAs and the resulting active consensus sunflower AW box motif can be used to summarize the coordinated co-regulation of the pathway by WRI1: blue background, WRI1–EMSA positive; red background, WRI1–EMSA negative; blue outline, WRI1–EMSA smear, probably positive; dashed-red outline, WRI1–EMSA smear, probably negative; solid red outline, AW box motif outside the 5′–UTR and predicted not to be bound by WRI1; orange background, absence of a WRI1 AW box motif; black outline, AW box motif not present in the CAS−6 line according to the Heliagene database.

WRI1 is described as a key regulator of genes involved in late glycolysis and FA biosynthesis in Arabidopsis seeds [11]. The activity of HaWRI1 as a transcriptional regulator of sunflower genes encoding enzymes involved in seed plastidial FA biosynthesis was confirmed in EMSAs using DNA fragments containing the consensus AW box motif found in the upstream regions of the sunflower genes studied: CNTNG(N)_7_CG [14]. These EMSA results indicate that sunflower WRI1 mainly drives and coordinates the early steps of FA synthesis in seed plastids (Figure 7). All of the subunits of the PDC complex are encoded by genes under the control of WRI1, as are the genes encoding for the αCT and BCCP subunits of the ACC complex. Later in the pathway, WRI1 regulated the transcription of two out of three ACP genes, and in the FAS complex, only the genes encoding KASIII, KASI and KAR activities clearly bound this transcription factor, unlike other FAS complex genes such as HAD or KASII. Interestingly, WRI1 also regulated the *FATA1* gene but no *FATB* gene, driving the sunflower seed toward the synthesis and export of oleic acid, although no SAD gene was found to be controlled by WRI1. This regulatory framework for FA synthesis in the seed plastid driven by HaWRI1 was consistent with that stated in the literature for Arabidopsis [10,12,14,19,20,23], as well as for *B. napus* [21] and other crops such as maize [18]. In Arabidopsis, a combination of microarrays, RT-PCR, yeast two-hybrid screening, EMSA and thermophoretic analysis, together with in vivo GUS experiments, established WRI1 target genes that included all the sunflower genes revealed here in EMSAs. Some of these, such as the KASI, ACP, *FATA* and *BCCP2* genes, were also described as candidate targets in *B. napus* or *Z. mays*. However, our confirmed candidates in sunflower did not include all of the targets described in Arabidopsis that are involved in plastidial FA biosynthesis, such as MCMT and HAD, as well as those involved in lipoic acid synthesis (LS, LT) and SAD genes [12,18,19,23,24]. MCMT is also described as a putative WRI1 target gene in *B. napus*, because it is upregulated in plants overexpressing *BnWRI1*, although neither this nor the HAD gene had an AW box in their upstream regions in the sunflower genome. 

Regarding sunflower LS, LT and SAD genes, some of their sunflower isoforms had AW box motifs in their upstream regions but outside of the 5′-UTR and far from the TIS, except for a putative motif at −42 bp for the *LT−5g* gene that was not detected in the genome of the sunflower CAS−6 line. The distance between the AW box and the TIS strongly influences the function of the AW box [14,20], and the majority of AW sites lie within 200 bp of the TIS in WRI1 target genes. Accordingly, when we tested the sunflower AW box motifs far from the TIS, including those in the SAD genes, they all failed to bind HaWRI1. Although multiple and diverse techniques have been used to identify the WRI1 target genes, the presence or absence of a specific target may reflect the technical sensitivity of these tests. In addition, we cannot rule out that the minor differences in WRI1 in terms of the regulation of FA synthesis in seed plastids may be specific to the plant species. Hence, the HAD, LS and SAD genes in another crop species such as maize are not ZmWRI1a target genes [18], as we described for sunflower.

Both the BC and ENR genes are described as AtWRI1 targets in binding assays of the AW box motif [14,23], yet it was unclear if they are HaWRI1 target genes based on the EMSA results alone, as a smear, not a clear band shift, was evident for both. However, the sequence analysis of their AW box motifs suggested that they may be regulated by WRI1, as they presented at least one AW box in their 5′-UTR that was consistent with our sunflower AW box consensus motif. Hence, the smeared lanes might be explained by less stable DNA binding, and thus, we should not rule them out as potential sunflower WRI1 targets. The in silico presence of an AW box motif in the sunflower *KAR1* upstream region but not in *KAR2* suggests that the *KAR1* gene but not *KAR2* is a putative WRI1 target in sunflower seeds [45]. The corresponding gene in Arabidopsis (*30AR*, *At1g24360*) is also a putative WRI1 target due to its upregulation in WRI1-overexpressed plants [14]. We amplified and sequenced the upstream regions of both sunflower genes, confirming the presence of the AW box motif in the *KAR1* upstream region but also detecting the presence of an almost identical sequence in the corresponding *KAR2*, albeit an AW box with an extra base. Unexpectedly, the non-canonical *KAR2* AW box motif was clearly bound by HaWRI1, as opposed to the *KAR1* canonical motif that produced a smear in the EMSA gel. Considering that the *KAR1* AW box conforms to the sunflower consensus motif, as occurred in the BC and ENR genes, and given the EMSA results for both genes, we propose that *KAR1* and *KAR2* are indeed HaWRI1 targets with possible differences in their affinity to the transcriptional regulator. Moreover, in vitro HaWRI1 binding to a non-canonical AW box opens the possibility of finding new WRI1 target genes and/or the number of binding sites in a promoter region. The two binding sites involved in sunflower KAR genes had almost identical sequences, yet EMSA with specific mutations at N positions revealed that base bias at N_2_ plays an important role in the binding stability between these cis elements and the regulator. Moreover, the selective in vitro binding of sunflower WRI1 to only some of the AW boxes tested suggests that WRI1 could act in a sequence-sensitive manner relative to the bases at N positions within the AW box, in addition to the *in vivo* position-sensitive binding described previously [20].

The AW box motif CNTNG(N)_7_CG was first described as the *cis* AtWRI1 binding element [14], and it was later studied in other plants such as *Z. mays* [18] and *B. napus* [21], confirming this motif as the consensus WRI1 binding motif in plants. Most previous studies focused on the presence/absence of this motif as a seal of the putative regulation of transcription by WRI1 when located close to the transcriptional or translational start site. In sunflower, we found this *cis* element in the upstream regions of seed plastidial FA synthesis genes, yet only 18 of 52 were recognized and bound by HaWRI1 in in vitro EMSAs. Most of the motifs not bound by WRI1 were located far from the TIS of the corresponding genes, but others were close to a TIS or at least within the 5′-UTR. Hence, the motif’s location is not sufficient to determine if WRI1 will bind to a motif, but probably, this also depends on the sequence itself, in which certain non-conservative positions play an important role. Our analysis of the sunflower AW box sequences recognized by HaWRI1 *in vitro* revealed some forbidden bases at specific N positions and a particularly relevant base bias for the N_2_ and N_6_ positions. From this analysis, a consensus sunflower AW box motif could be drafted as an active *cis* element involved in the regulation of FA biosynthetic gene transcription by WRI1 in seed.

These active sunflower seed motifs were mainly found as single binding boxes in the gene’s promoter region, and only two genes were found to have two active AW boxes in their upstream regions, *LPD−10g* and *BCCP−16g*. In Arabidopsis, the 5′-UTR of *BCCP2* contains two AW boxes, and expression of the *BCCP2* gene is much more strongly induced by WRI1 relative to genes with a single AW box [14]. According to the transcriptomic data for the common sunflower (Heliagene), the *LPD−10g* and *BCCP−16g* genes were expressed strongly in seeds, with only a slightly higher number of transcripts in ligules and roots, respectively, yet at a similar level to other genes that have only a single AW box, *LPD−16g*, *BCCP−2g* and *BCCP−9g*. Thus, there may be other factors playing a role in regulating the transcription of genes involved in FA synthesis in sunflower seeds. Nevertheless, the active motifs identified did indicate that the PDC (LPD, β-PDH), ACC (BCCP) and KASIII gene families expressed strongly in sunflower seeds contain active AW motifs in their promoter regions (genes that clearly shifted in EMSAs). By contrast, *LPD−5g* and *BCCP−5g* had no AW motifs, and they were expressed weakly in seeds compared to other members of their families. The β-PDH and KASIII genes all contain AW boxes and are expressed more strongly in seeds when they contain an active motif, such as *β-PDH−16g* and *β-PDH−17g* as opposed to *β-PDH−4g*, and *KASIII−2g* and *KASIII−5g* as opposed to *KASIII−17g*. However, it does not appear to be a general rule, as the ACP gene expressed most strongly in seeds was that with no AW motif in its promoter region, the *ACP−8g* (*ACP3*) gene. These results confirmed that while other regulatory factors might be active in these events, the use of WRI1 to regulate FA synthesis in sunflower seed plastids focuses mainly on early stages of the pathways involved, those of acetyl-CoA and malonyl-CoA synthesis by the PDC and ACC complexes, respectively, together with the first step in the FAS complex, 3-ketoacyl-ACP synthesis by KASIII. Moreover, the fact that the single gene coding for *HaFATA1* was the only thioesterase gene bound by HaWRI1 in EMSA, and none of the FATB genes were retarded by this transcription factor (*FATB1* or *FATB−9g*), leads us to propose a push–pull strategy employed by sunflower WRI1 to regulate seed plastidial FA synthesis, directing it toward the synthesis and export of oleic acid.

## 4. Conclusions

Sunflower WRI1 recognizes and binds target genes of the FA synthesis, confirming a coordinated regulation of the pathway in seed plastids, as previously and extensively described in Arabidopsis, with the main particularity of a push–pull strategy that stimulates the synthesis and export of oleic acid to the cytosol. Furthermore, the leading novelties of this work are the finding of the sequence-sensitive binding of WRI1 to its targets, in which the non-conservative bases of the AW box play a key role, and the discovery of a non-consensus AW box also recognized by WRI1. Our work is limited to the sunflower, but it opens the door for considering non-consensus AW boxes for WRI1 binding or the number of sites in a target promoter.

## 5. Materials and Methods

### 5.1. Plant Material and Growth Conditions

Sunflower seeds from the common CAS−6 sunflower line (RHA−274 genetic background) were germinated in wet perlite at 25 °C and then moved to a germination chamber for 2 weeks. Subsequently, the seedlings were transferred to growth chambers and grown on 25 °C/15 °C (day/night) cycles in bags endowed with fertilizer. They were grown on a 16 h photoperiod with a photon flux density of 250 μmol m^−2^ s^−1^.

Genomic DNA from mature sunflower leaves was extracted according to the modified cetyl-trimethyl ammonium bromide (CTAB) method [57]. Total RNA was extracted from 21 days-after-flowering (DAF) sunflower seeds using the Spectrum Plant Total RNA Kit (Sigma-Aldrich, St. Louis, MO, USA), according to the manufacturer’s instructions. The total RNA obtained (1 μg) was used to synthesize cDNA using the Ready-To-Go T-Primed First Strand Kit (Amersham Bioscience, Roosendaal, The Netherlands).

### 5.2. HaWRI1-DNA Binding Domain Cloning, Expression in Escherichia coli and Purification

The coding sequence of WRINKLED1 from the CAS−6 line (*HaWRI1*) was amplified by PCR from a cDNA pool of 21 DAF seeds using the HaWRI1-F and HaWRI1-R primer pair (Table S1). The cDNA obtained was cloned into the pMBL-T vector (CANVAX Biotech, Córdoba, Spain), and its sequence was verified by DNA sequencing and deposited at GenBank (Acc. Number JX424422.1).

A truncated version of *HaWRI1* encoding the WRI1-DNA binding domain (HaWRI1_DBD, amino acids 51 to 229), was cloned into the *pET-trx1a* expression vector (Baud et al., 2009) to obtain a 6-His-TRX-WRI1_DBD fusion protein. HaWRI1_DBD was amplified with the Phusion High-Fidelity DNA polymerase (Thermo Fisher Scientific, Waltham, MA, USA) using the previously cloned full length HaWRI1 cDNA as a template, and the HaWRI1-DBD-F and HaWRI1-DBD-R primers (Table S1). The PCR product was digested and cloned into the *pET-trx1a* vector as a *Nco*I-*Xho*I fragment, removing the GFP coding sequence from this vector. The empty *pET-trx1a* vector provided the 6-His-TRX-GFP construct used in this work. The 6-His-TRX construct was obtained after digesting the *pET-trx1a* plasmid with the *Nco*I and *Xho*I restriction endonucleases (New England Biolabs, Hitchin, UK), removing the cDNA insert corresponding to the GFP coding sequence. After blunting the plasmid ends with Klenow enzyme (New England Biolabs, Hitchin, UK), the plasmid was re-ligated with the T4 DNA Ligase (Thermo Fisher Scientific, Waltham, MA, USA). All plasmids used here were verified by DNA sequencing.

Truncated protein expression and purification were carried out as described previously [11], with minor modifications in the dialysis step. The fractions containing the protein were pooled, the imidazole was removed on a PD−10 (sephadex G−25) column (GE Healthcare, Chicago, IL, USA), and the protein was concentrated in a centrifugal concentrator (Amicon MWCO 3 kDa: Merck, Darmstadt, Germany) and stored at −20 °C in dialysis buffer (150 mM NaCl, 20 mM Tris-HCl [pH 8.0], 2 mM MgCl_2_, 0.25 mM EDTA, 0.02% Nonidet P−40 and 20% glycerol). The purity of the recombinant protein was evaluated by SDS-PAGE and Coomassie staining (ChemiDoc Imaging System; BioRad, Hercules, CA, USA), and the concentration of the purified recombinant protein was determined with a Bio-Rad protein assay kit using bovine serum albumin (BSA) as a standard. Recombinant 6-His-TRX and 6-His-TRX-GFP proteins (used as a negative EMSA binding control), were obtained by the same procedure but starting from *E. coli* cells containing the 6-His-TRX construct or empty *pET-trx1a* vector, respectively.

### 5.3. Promoter Cloning, DNA Fragment Amplification and Purification, and Site-Directed Mutagenesis by Overlap Extension PCR

All of the oligonucleotide sequences used in this work are shown in Table S1 and were designed with the help of applications available online: *primer3 4.0* (https://bioinfo.ut.ee/primer3-0.4.0/. Accessed on 11 January 2021) and *OligoAnalyzer Tools* (https://eu.idtdna.com/pages/tools/oligoanalyzer. Accessed on 11 January 2021). Oligonucleotide synthesis and DNA sequencing were performed by Eurofins Genomics (https://eurofinsgenomics.eu. Accessed on 19 April 2021).

The promoter regions of the sunflower *FATA1*, *FATB1*, *SAD6*, *SAD17*, *KAR1* and *KAR2* genes (GenBank Acc. Numbers AY078350, AF036565, U91339.1, U91340, HM021135 and HM021136, respectively) were amplified by PCR from sunflower CAS−6 genomic DNA using the Phusion High-Fidelity DNA polymerase (Thermo Fisher Scientific, Waltham, MA, USA), and they were cloned into the *pMBL-T* vector (CANVAX Biotech, Córdoba, Spain). All of the cloned promoter regions were located upstream of the TIS. The promoter length and the oligonucleotide pairs used for cloning are described in Tables S2 (FAT and SAD genes) and 4 (KAR genes), and all of the DNA clones obtained were verified by sequencing.

The promoter DNA fragments used here in the EMSAs were situated within −500 bp of the TIS of their corresponding gene, with some exceptions. Each DNA fragment was amplified by PCR from sunflower CAS−6 genomic DNA or the previously cloned promoter region as the template, using the Phusion High-Fidelity DNA polymerase (Thermo Fisher Scientific, Waltham, MA, USA) and the corresponding pair of oligonucleotides (see primer pairs used in Tables S3 and S4, together with the product lengths and their location upstream of the TIS). PCR products were purified from agarose gels using the ISOLATE II PCR and Gel Kit (Bioline, Memphis, TN, USA), and quantified in a NanoDrop One C Spectrophotometer (Thermo Scientific, Waltham, MA, USA). All EMSA DNA PCR fragments were confirmed by sequencing.

Site-directed mutagenesis was performed by overlap extension PCR [58] to introduce selected point mutations or DNA deletions into upstream sunflower DNA regions. The DNA fragment containing each wild type AW box sequence was used as the template for the first double PCR, and using the P1/P2 and P3/P4 oligonucleotide pairs, respectively, these containing the desired point mutation or deletion. The PCR products obtained were diluted 1/100, mixed and used as the template for extension and amplification in a second PCR with the P1/P4 oligonucleotide pair. The final PCR products consisted of the desired EMSA DNA fragments containing the mutated or deleted AW boxes. The correct introduction of the mutations was confirmed by sequencing (see Table S5 for the mutations introduced, the EMSA DNA fragments generated and the mutagenic primer pairs used in each PCR -P1/P2, P3/P4, P1/P4).

### 5.4. Agarose Gel Electrophoretic Mobility Shift Assays (EMSAs)

Different amounts of the recombinant HaWRI1_DBD fusion protein (6-His-TRX tagged, 0–640 ng range) were incubated for 30 min at room temperature with a single promoter DNA fragment (300 ng) in 20 μL of binding buffer (20 mM Tris-HCl [pH 8.0], 250 mM NaCl, 2 mM MgCl_2_, 1% glycerol, 1 mM DTT and 1 mg/mL BSA). Samples were then loaded onto a 1% agarose gel and resolved for 90 min at 4 °C at 100 V in 1× TAE buffer (40 mM Tris-HCl [pH 8.0], 20 mM acetic acid and 1 mM EDTA). The gel was soaked in 1× TAE buffer containing 0.5 μg/mL RedSafe (INtRON Biotechnology, Burlington, MA, USA) for 30 min and then visualized in a UV transilluminator. For each analysed DNA promoter fragment, EMSAs were performed an average of 3–5 times.

### 5.5. Acrylamide Gel Electrophoretic Mobility Shift Assays (EMSAs)

The f1, f2 and f3 DNA fragments that corresponded to the *FATA1* promoter region were designed to cover a 1 kb stretch relative to the ATG start codon in three overlapping fragments, each around 300−400 bp in length: f1, from −272 bp to +51 bp; f2, from −567 bp to −232 bp; and f3, from −918 bp to −534 bp. The *FATA1* promoter DNA fragment corresponding to f1 but without the AW box (*f1-ΔAWbox*, 299 bp) was generated by overlap extension PCR using the oligonucleotide pairs described in Table S5 (see above). A 24 bp DNA fragment containing the 14 bp AW box sequence present in *FATA1* f1 was obtained by room temperature hybridization of the two complementary oligonucleotides pHaFATA1-AWbox-F and pHaFATA1-AWbox-R (Table S1).

EMSA DNA fragments were labelled with digoxigenin (DIG) following the manufacturer’s instructions (DIG Gel Shift Kit 2nd Generation; Roche Diagnostics, Indianapolis IN, USA), and 30 fmol of labelled DNA was incubated with different amounts of purified HaWRI1_DBD recombinant protein (0–1000 ng) in 20 µL of binding buffer plus 0.05 µg/µL polydIdC. For competition assays, the unlabelled competitor was incubated with the protein briefly before adding the labelled DNA, and after adding the labelled DNA the reactions were incubated for 30 min at room temperature. The binding reactions were fractionated at 4 °C by electrophoresis at 80 V for 120 min on 6% native polyacrylamide gels (PAGE) in 0.5X TBE buffer (44.5 mM Tris-HCl [pH 8.0], 44.5 mM boric acid, 1 mM EDTA [pH 8.0]). Following electrophoretic separation, the oligonucleotide-protein complexes were transferred to a positively charged nylon membrane (Hybond-N+; Cytiva, Marlborough, MA, USA), and the DIG labelled DNA was visualized in an enzyme immunoassay using anti-Digoxigenin-AP and a chemiluminescent substrate, following the manufacturer’s instructions. The chemiluminescent signals generated were recorded on an imaging device (ChemiDoc Imaging System; BioRad, Hercules, CA, USA). 

### 5.6. Bioinformatics Analysis

Protein sequences from known Arabidopsis genes involved in FA biosynthesis (http://aralip.plantbiology.msu.edu/pathways/fatty_acid_synthesis) [3] were obtained from The Arabidopsis Information Resource, TAIR, (https://www.arabidopsis.org/. Accessed on 12 November 2020) [59]. These sequences were used to search for homologous genes in the sunflower genome and to extract their promoter regions and transcriptomic data using the tools available in RELEASE 2.0 (v2020) of the Sunflower genome portal (Heliagene; INRA Sunflower Bioinformatic Resources, https://www.heliagene.org/. Accessed on 12 January 2021) [60]. The in silico localization of these proteins was predicted using DeepLoc (http://www.cbs.dtu.dk/services/DeepLoc/. Accessed on 3 February 21) [61] and Localizer (http://localizer.csiro.au/. Accessed on 3 February 21) [62] tools.

The DNA promoter regions were scanned to identify and localize AW box sequences (according to Maeo et al. [14]) using the PlantPAN 3.0 tool (http://plantpan.itps.ncku.edu.tw/. Accessed on 3 February 2021) [63]. The Motif Alignment and Search Tool (MAST–http://meme-suite.org. Accessed on 7 May 2021) [64] and WebLogo (https://weblogo.berkeley.edu/logo.cgi. Accessed on 7 May 2021) [65] were used to obtained the consensus motif for the sunflower AW box.

## Figures and Tables

**Table 1:** WRI1-binding motifs (AW box: CNTNG(N)_7_CG) and their position relative to the ATG codon in the promoter regions of sunflower Acyl–ACP thioesterases (*FATA1*; *FATB1*) and stearoyl-ACP desaturases (*SAD6*; *SAD17*). The asterisk marks a non-canonical sequence due to the appearance of an extra base (in bold) in the only motif found in *SAD6* similar to an AW box. The sunflower genes are named according to previous publications, followed by the chromosome number, as stated in this work for other sunflower genes: TIS, Translational Initiation Site (ATG codon).

Heliagene ID (v2020)	Sunflower Gene	Strand	AW Box Sequence (5′−3′)	AW Box Position (bp from the TIS)
*HanXRQr2Chr01g0022981*	*FATA1−1g*	+	CCTCGTGCATATCG	−110/−97
		+	CGTCGCGTCGCGCG	−900/−887
*HanXRQr2Chr06g0263811*	*FATB1−6g*	+	CGTCGCTGTTTCCG	+23/+36
		−	CCTTGCTTGCCTCG	−1419/−1406
*HanXRQr2Chr01g0035021*	*SAD17−1g*	−	CCTTGTGAGAATCG	−610/−597
		−	CCTTGATATCATCG	−1159/−1146
*HanXRQr2Chr11g0503601*	*SAD6−11g*	+	CATAGGCAACTTACG *	−670/−656

**Table 2:** Sunflower genes involved in plastidial fatty acid biosynthesis. Arabidopsis genes were used as queries to search for sunflower homologs in a public database (Heliagene). The sunflower gene names are those taken from Heliagene ID (v2020), with a shorter name used here according to the gene function acronym followed by the number of chromosome location: # Heliagene ID (v2018). Gene names already described in the literature are indicated in brackets. The AW box motifs were found with the PlantPAN 3.0 tool. The subcellular localization was defined with the DeepLoc1.0 and Localizer free online applications (in case of discrepancy, if one of the two programs indicated a plastidial/chloroplastic location, this was the location selected to appear in Table 2, and the gene was still in our study). Gene expression in sunflower seeds is indicated according to the transcriptomic data from Heliagene–v2018 as YES (Y), indicating the transcript quantity as reads per kilobase per million (rpkm) when available (Heliagene–v2020). Bibliographic references are given when seed expression has previously been described in the CAS−6 line.

	Plastidial Fatty Acid Synthesis Gene	Arabidopsis Gene	Sunflower Gene Heliagene ID-v2020	Sunflower Gene Shorter Name	Number of AW Boxes	Subcelullar Localization	Gene Expression in SunFlower Seed Y/N-rpkm (Heliagene)	Gene Expression in SunFlower Seed (CAS-6 Line)
PDC	*α-PDH*	*At1g01090*	HanXRQr2Chr13g0619071	α-PDH-13g	1	Plastid	Y	
	*β-PDH*	*At1g30120*	HanXRQr2Chr04g0172911	β-PDH-4g	1	Plastid	Y-0.02	
			HanXRQr2Chr16g0768891	β-PDH-16g	2	Plastid	Y-55	
			HanXRQr2Chr17g0823581	β-PDH-17g	3	Plastid	Y-35	
	*LPD*	*At3g16950*	HanXRQr2Chr05g0217331		0	Plastid	Y-2	
			HanXRQr2Chr10g0440741	LPD-10g	2	Plastid	Y-13	
			HanXRQr2Chr16g0749581	LPD-16g	2	Plastid	Y-11	
	*DHLAT*	*At3g25860*	HanXRQr2Chr04g0156631	DHLAT-4g	2	Plastid	Y-35	
			HanXRQr2Chr08g0324561	DHLAT-8g	1	Plastid	Y-30	
			HanXRQr2Chr12g0535191	DHLAT-12g	1	Plastid	Y	
ACC	*α-CT*	*At2g38040*	HanXRQr2Chr10g0429681	α-CT-10g	4	Plastid	Y-3	
			HanXRQr2Chr10g0429871		0	Plastid	Y	
			HanXRQr2Chr15g0671211	α-CT-15g	2	Plastid	Y-5	D.N.S.
	*β-CT*	*AtCg00500*	HanXRQr2CPg0836391		0	Plastid	Y-0.8	D.N.S.
	*BC*	*At5g35360*	HanXRQr2Chr16g0770591	BC-16g	6	Plastid	Y-35	D.N.S.
			HanXRQr2Chr17g0824731		0	Plastid	Y-7	
	*BCCP*	*At5g15530*	HanXRQr2Chr02g0061841	BCCP-2g	1	Plastid	Y-18	D.N.S.
			HanXRQr2Chr05g0231761		0	Plastid	Y-3	
			HanXRQr2Chr09g0408331	BCCP-9g	2	Plastid	Y-85	
			HanXRQr2Chr16g0729021	BCCP-16g	6	Plastid	Y-23	D.N.S.
	*MCMT*	*At2g30200*	HanXRQr2Chr01g0018151		0	Plastid	Y-51	
			HanXRQr2Chr01g0018201		0	Plastid	Y-0.05	
	*HACPS*	*At3g11470*	HanXRQr2Chr04g0161011	HACPS-4g	0	Mitochondrion	Y-1	
			HanXRQr2Chr07g0300321	HACPS-7g	1	Mitochondrion	Y-1.6	
	*ACP*	*At4g25050*	HanXRQr2Chr07g0290651 (*ACP1*)	ACP-7g	2	Plastid	Y-0.11	[40]
			HanXRQr2Chr08g0343921 (*ACP3*)		0	Plastid	Y-370	[40]
			HanXRQr2Chr09g0412861		0	Plastid	Y	
			HanXRQr2Chr14g0647771 (*ACP2*)	ACP-14g	6	Plastid	Y-1.5	[40]
			HanXRQr2Chr17g0819021		0	Mitochondrion	Y-8	
FAS	*KASI*	*At5g46290*	HanXRQr2Chr01g0024181	KASI-1g	1	Plastid	N.D.	
			HanXRQr2Chr01g0027641		0	Plastid	N.D.	
			HanXRQr2Chr04g0179841		0	Plastid	Y-38	
			HanXRQr2Chr13g0603901		0	Plastid	Y-0.1	
			HanXRQr2Chr17g0822771	KASI-17g	1	Plastid	Y-90	
			HanXRQr2Chr17g0780601		0	Peroxisome	N.D.	
	*KASII*	*At1g74960*	HanXRQr2Chr06g0259761	KASII-6g	2	Peroxisome	N.D.	
			HanXRQr2Chr09g0361681		0	Plastid	Y-3	
			HanXRQr2Chr15g0722271	KASII-15g	2	Plastid	Y-1	
	*KASIII*	*At1g62640*	HanXRQr2Chr02g0073211 (*KASIII*)	KASIII-2g	3	Plastid	Y-50	[46]
			HanXRQrChr05g0148701#	KASIII-5g	1	Plastid	Y-55	
			HanXRQr2Chr17g0801431	KASIII-17g	3	Plastid	Y-5	
	*KAR*	*At1g24360*	HanXRQr2Chr10g0464751 (*KAR2*)		0	Plastid	Y	[45]
			HanXRQr2Chr17g0781961 (*KAR1*)	KAR-17g	1	Plastid	Y-100	[45]
	*HAD*	*At2g22230*	HanXRQr2Chr03g0102091 (*HAD2*)		0	Plastid	Y-25	[44]
			HanXRQr2Chr10g0443511 (*HAD1*)		0	Plastid	Y-0.03	[44]
	*ENR*	*At2g05990*	HanXRQr2Chr02g0082991 (*ENR1*)		0	Plastid	Y-7	[43]
			HanXRQr2Chr16g0768471 (*ENR2*)	ENR-16g	3	Plastid	Y-20	[43]
			HanXRQr2Chr17g0822551	ENR-17g	3	Plastid	Y-28	
	*LS*	*At5g08415*	HanXRQr2Chr05g0201611 (*LIP1p1*)	LS-5g	1	Plastid	Y-1.5	[38]
			HanXRQr2Chr12g0526011 (*LIP1p2*)	LS-12g	1	Plastid	Y-0.02	[38]
	*LT*	*At4g31050*	HanXRQr2Chr05g0205061 (*LIP2p*)	LT-5g	1	Plastid	Y-4	
			HanXRQr2Chr09g0406031		0	Plastid	N.D.	
FAT	*FATA*	*At3g25110*	HanXRQr2Chr01g0022981 (*FATA1*)	FATA-1g	2	Plastid	Y-73	[41]
	*FATB*	*At1g08510*	HanXRQr2Chr05g0207531		0	Plastid	Y-1	
			HanXRQr2Chr06g0263811 (*FATB1*)	FATB-6g	2	Plastid	Y-0.02	[42]
			HanXRQr2Chr09g0365831	FATB-9g	3	Plastid	Y-13	D.N.S.
	*SAD*	*At2g43710*	HanXRQr2Chr01g0035021 (*SAD17*)	SAD-1g	2	Plastid	Y-310	
			HanXRQr2Chr11g0503601 (*SAD6*)		0	Plastid	Y-120	

Abbreviations: PDH, Pyruvate dehydrogenase; DHLAT, Dihydrolipoamide acetyl transferase; LPD, Dihydrolipoamide dehydrogenase; BC, Biotin carboxylase; BCCP, Biotin carboxyl carrier protein; CT, Carboxyltransferase; MCMT, Malonyl–CoA–ACP malonyl transferase; HACPS, Holo–ACP–synthase; KAR, β–ketoacyl–ACP reductase; HAD, Hydroxyacyl–ACP dehydrase; KAS, Ketoacyl–ACP synthase; ENR, Enoyl–ACP reductase; ACP, Acyl carrier protein; LS, Lipoate synthase; LT, Lipoyltransferase; FAT, acyl–ACP thioesterase; SAD, Stearoyl–ACP desaturase; N.D., No Data; D.N.S., Data not shown.

**Table 3:** AW box motifs found in the promoter regions of sunflower genes probably involved in plastidial fatty acid biosynthesis. A search for the AW box motif was carried out using the PlantPAN 3.0 tool. The gene name is stated according to the functional acronym and chromosome location, and the position of the AW box motif is indicated as initial/end bp from the TIS and labelled with a YES (Y) when it lies within the 5′–UTR. Sunflower genes already described in the literature are underlined. The sequences selected for EMSA are highlighted: orange (located within 5′–UTR); blue (located outside the 5′–UTR); # indicates that the DNA sequence presents in *KAR2* promoter containing an extra base (underlined in bold) within the AW box motif. Point mutations in the *KAR*’s AW box motif are underlined in bold.

Sunflower Gene	Strand	AW Box Sequence (5′-3′)	AW Box Position (bp from the TIS)	AW Box in the 5′-UTR	Sunflower Gene	Strand	AW Box Sequence (5′-3′)	AW Box Position (bp from the TIS)	AW Box in the 5′-UTR
*αPDH-13g*	+	CCTCGTTCACTCCG	−101/−88	Y	*ACP1* *-7g*	+	CATTGTACCCTCCG	−40/−27	Y
*βPDH-4g*	−	CTTCGATTGTGCCG	−168/−155	Y		−	CATCGGGATATCCG	−126/−113	Y
*βPDH-16g*	−	CTTTGGTTGTAACG	−179/−166	Y	*ACP2* *-14g*	+	CATCGTCTCCTCCG	−65/−52	Y
	−	CATTGCGTTTTACG	−958/−944			−	CATCGAGAAATCCG	−124/−110	Y
*βPDH-17g*	+	CATTGATTTCTCCG	−99/−86	Y		+	CGTCGTTGCCGACG	−859/−846	
	−	CATAGTAACACCCG	−276/−263			−	CGTCGGCAACGACG	−859/−846	
	−	CGTGGGCGTCATCG	−464/−451			−	CCTGGGTGATAACG	−882/−869	
*LPD-10g*	−	CTTCGAGAGGTCCG	−73/−60	Y		−	CGTCGTTTCCATCG	−1195/−1182	
	+	CATTGTTAACTCCG	−98/−85	Y	*KASI-1g*	+	CGTTGTTATTTACG	−991/−978	
*LPD-16g*	+	CGTCGTCTCCCCCG	−112/−99	Y	*KASI-17g*	+	CATCGTTTATATCG	−101/−87	Y
	+	CTTCGTTAACTCCG	−127/−114	Y	*KASII-15g*	+	CCTGGAAACAAACG	−1240/−1226	
*DHLAT-4g*	−	CGTTGGTTAGCGCG	−641/−628			+	CTTTGGGTTTTTCG	−1290/−1277	
	−	CGTCGTATCTACCG	−675/−622		*KASIII* *-2g*	−	CATTGAGAAGAACG	−71/−58	Y
*DHLAT-8g*	−	CTTAGGCCTTAACG	−703/−690			−	CTTCGGAGTAGTCG	−109/−96	Y
*DHLAT-12g*	+	CCTCGTCTTCTCCG	−105/−92	Y		+	CTTCGACTACTCCG	−112/−99	Y
*αCT-10g*	−	CATAGAGAAGATCG	−915/−902	Y	*KASIII-5g*	+	CCTCGACTACTCCG	−209/−196	Y
	+	CATAGAACTCATCG	−1095/−1082		*KASIII-17g*	+	CTTGGAGTACTCCG	−277/−264	
	−	CGTCGAAAATCCCG	−1383/−1370			+	CGTGGTGTCTACCG	−342/−329	
	−	CTTGGAATTTTTCG	−1406/−1393			+	CCTTGAGCCAAACG	−376/−363	
*αCT-15g*	−	CATAGAGAAGATCG	−870/−857	Y	*KAR1* *-17g*	+	CTTAGATTATATCG	−130/−117	Y
	−	CATGGGTGGGTTCG	−995/−982	Y	*mut A/G*		CTTAGGTTATATCG		
*BC-16g*	+	CATAGATTTCATCG	−334/−321	Y	*mut A/C*		CTTCGATTATATCG		
	+	CGTTGTCTAAATCG	−1042/−1029		*KAR2* *-10g*	+	CTTTAGGTTATATCG #	−153/−139	
	−	CGTCGAAAATCCCG	−1217/−1204		*mut T/C*		CTTCAGGTTATATCG		
	−	CTTGGAATTTTTCG	−1240/−1227		*ENR2* *-16g*	+	CATCGTTTTGATCG	−997/−984	Y
	−	CCTTGAATTTTTCG	−1262/−1249			−	CTTTGTGAGGGCCG	−1020/−1007	Y
	−	CTTGGAATTTTTCG	−1285/−1272			−	CTTTGGAAGTATCG	−1042/−1029	Y
*BCCP-2g*	+	CTTTGTTGATATCG	−104/−91	Y	*ENR-17g*	+	CTTTGTTTTAATCG	−644/−631	Y
*BCCP-9g*	+	CCTCGGTCATATCG	−123/−110	Y		−	CTTTGTGAGGGCCG	−677/−664	Y
	−	CTTTGTTACAGGCG	−1004/−991			−	CTTTGGAAGTATCG	−699/−686	Y
*BCCP-16g*	+	CTTCGGTTCCCTCG	−92/−79	Y	*FATA1* *-1g*	+	CCTCGTGCATATCG	−110/−97	Y
	+	CCTCGTTGATATCG	−136/−123	Y		+	CGTCGCGTCGCGCG	−900/−887	
	−	CCTCGTGTGTCACG	−252/−239	Y	*FATB1* *-6g*	−	CCTTGCTTGCCTCG	−1419/−1406	
	+	CATCGCATAAACCG	−1048/−1035		*FATB-9g*	−	CGTTGAACGGAACG	−2174/−2161	
	+	CTTCGAATAAATCG	−1203/−1190			−	CTTCGTTTGGATCG	−2357/−2344	
	−	CTTAGGCGACAGCG	−1242/−1229			−	CTTCGTCTCCGTCG	−2375/−2362	
*(LS) LIP1p1-5g*	−	CCTTGTCAAGTACG	−867/−854		*SAD17* *-1g*	−	CCTTGTGAGAATCG	−610/−597	
*(LS) LIP1p2-12g*	−	CATGGCTGTCTACG	−570/−557			−	CCTTGATATCATCG	−1159/−1146	
*(LT) LIP2p-5g*	+	CTTCGGTCACTCCG	−42/−23	Y					

Abbreviations: PDH, Pyruvate dehydrogenase; DHLAT, Dihydrolipoamide acetyl transferase; LPD, Dihydrolipoamide dehydrogenase; BC, Biotin carboxylase; BCCP, Biotin carboxyl carrier protein; CT, Carboxyltransferase; KAR, β–ketoacyl–ACP reductase; KAS, Ketoacyl–ACP synthase; ENR, Enoyl–ACP reductase; ACP, Acyl carrier protein; LS, Lipoate synthase; LT, Lipoyltransferase; FAT, acyl–ACP thioesterase; SAD, Stearoyl–ACP desaturase; TIS, Translational Initiation Site.

## Data Availability

Not applicable.

## Supplementary Materials

The following supporting information can be downloaded at: https://www.mdpi.com/article/10.3390/plants11070972/s1, Figure S1: SDS-PAGE followed by Coomassie staining to show the purified recombinant proteins used in EMSA; Figure S2: HaWRI1_DBD binding to DNA fragment 1 of the sunflower *FATA1* promoter region (*pHaFATA1-f1*) containing an AW box motif and specific control binding in Digoxigenin (DIG) labelled EMSA; Figure S3: HaWRI1_DBD binding to the sunflower β-ketoacyl-ACP reductase (*KAR1* and *KAR2*) promoter regions and specific control binding reactions in agarose EMSA; Table S1: Oligonucleotides used in this work; Table S2: Promoter regions of the sunflower FAT and SAD genes described in the literature; Table S3: EMSA DNA fragments from sunflower FAT and SAD promoter regions and control genes; Table S4: EMSA DNA fragments from upstream regions of sunflower genes studied here that are involved in plastidial fatty acid biosynthesis; Table S5: EMSA DNA fragments from sunflower genes obtained by site-directed mutagenesis overlap extension PCR.
