# Supplementary material for: The Sunflower WRINKLED1 Transcription Factor Regulates Fatty Acid Biosynthesis Genes through an AW Box Binding Sequence with a Particular Base Bias
Source: Plants (Basel). 2022 Apr 2;11(7):972. doi: 10.3390/plants11070972 (PMC9002759; doi:10.3390/plants11070972)

Figure S1. SDS-PAGE followed by Coomassie staining to show the purified recombinant proteins used in EMSA. *Ha*WRI1_DBD, sunflower 6-His-Thioredoxin-WRINKLED1_DNA Binding Domain (312 aa, 35 kDa); TRX, 6-His-Thioredoxin fusion protein (163 aa, 18 kDa); GFP, 6-His-Thioredoxin-Green Fluorescent Protein (773 aa, 41 kDa). MW, Molecular Weight; T, total fraction; SB, soluble fraction; P, purified protein.

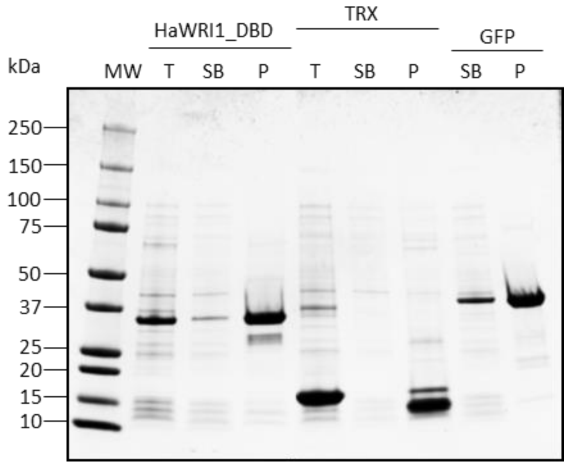

Supplement: Supplementary file 1 [file plants-11-00972-s001.zip › plants-1663482-supplementary/Suppl Files/Supplementary Figure S1.pdf]
