# Supplementary material for: The Sunflower WRINKLED1 Transcription Factor Regulates Fatty Acid Biosynthesis Genes through an AW Box Binding Sequence with a Particular Base Bias
Source: Plants (Basel). 2022 Apr 2;11(7):972. doi: 10.3390/plants11070972 (PMC9002759; doi:10.3390/plants11070972)

Figure S3. *Ha*WRI1_DBD binding to the sunflower β-ketoacyl-ACP reductase (*KAR1* and *KAR2*) promoter regions and specific control binding reactions in agarose EMSA. The arrow shows the DNA shifted by WRI1 binding, the DNA (300 ng) consisted of the corresponding promoter region containing the AW box motif studied, as indicated by its location from ATG codon: WRI1 (W), 6-His-TRX-WRI1_DBD fusion protein (150-300-600 ng); TRX (T), 6-His-Thioredoxin protein (150-300-600 ng); GFP (G), 6-His-TRX-Green Fluorescent protein (150-600 ng); uDNA1, non-specific DNA1 (*HacPGK2*); uDNA2, non-specific DNA2 (*HaCWI3*). The sunflower KARs are named according to previous publications and they are followed by the chromosome number where they are located.

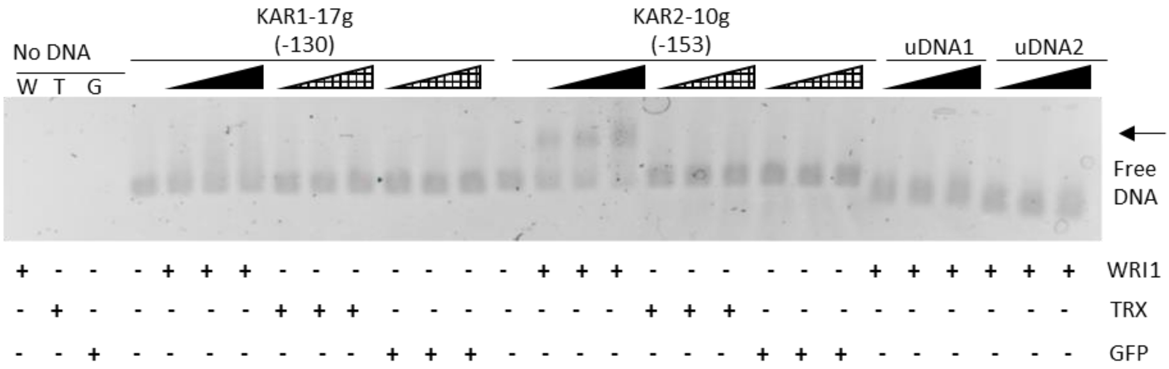

Supplement: Supplementary file 1 [file plants-11-00972-s001.zip › plants-1663482-supplementary/Suppl Files/Supplementary Figure S3.pdf]
