# Supplementary material for: The Sunflower WRINKLED1 Transcription Factor Regulates Fatty Acid Biosynthesis Genes through an AW Box Binding Sequence with a Particular Base Bias
Source: Plants (Basel). 2022 Apr 2;11(7):972. doi: 10.3390/plants11070972 (PMC9002759; doi:10.3390/plants11070972)

Figure S2. *Ha*WRI1_DBD binding to DNA fragment 1 of the sunflower *FATA1* promoter region (*pHaFATA1-f1*) containing an AW box motif and specific control binding in Digoxigenin (DIG) labelled EMSA. The arrow shows the DNA shifted by WRI1 binding. DIG labeled or unlabeled (■ cold competitor reaction) dsDNA (30 fmol): f1 (323 bp) and f1-ΔAWbox (299 bp) with or without the 14 bp AW box motif, respectively. WRI1, 6-His-TRX-WRI1_DBD fusion protein (◢ 160-800 ng); GFP, 6-His-TRX-GFP fusion protein (◢ 160-800 ng); TRX, 6-His-Thioredoxin protein (◢ 160-800 ng).

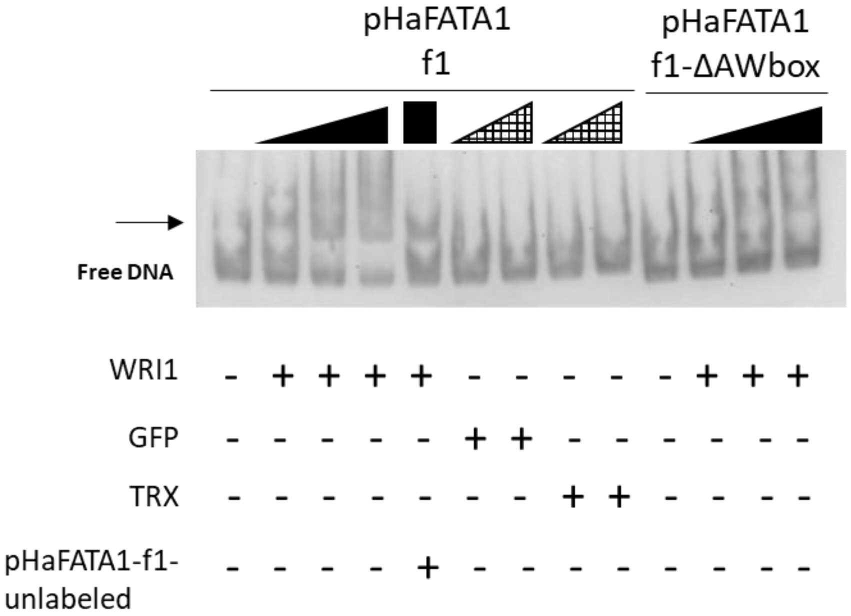

Supplement: Supplementary file 1 [file Supplementary Figure S2.pdf › plants-1663482-supplementary/Suppl Files/Supplementary Figure S2.pdf]
